# Stability of the Darwinian Dynamics: Effect of Intraspecific Competition and Human Intervention

**DOI:** 10.1007/s13235-025-00629-3

**Published:** 2025-03-20

**Authors:** Mohammadreza Satouri, Jafar Rezaei, Kateřina Staňková

**Affiliations:** Faculty of Technology, Policy and Management, Institute for Health Systems Science, Delft University of Technology, Delft, The Netherlands

**Keywords:** Evolutionary game theory, Eco-evolutionary dynamics, Darwinian dynamics, Stability, Mathematical oncology

## Abstract

We analyze the stability of a game-theoretic model of a polymorphic eco-evolutionary system in the presence of human intervention. The goal is to understand how the intensity of this human intervention and competition within the system impact its stability, with cancer treatment as a case study. In this case study, the physician applies anti-cancer treatment, while cancer, consisting of treatment-sensitive and treatment-resistant cancer cells, responds by evolving more or less treatment-induced resistance, according to Darwinian evolution. We analyze how the existence and stability of the cancer eco-evolutionary equilibria depend on the treatment dose and rate of competition between cancer cells of the two different types. We also identify initial conditions for which the resistance grows unbounded. In addition, we adopt the level-set method to find viscosity solutions of the corresponding Hamilton–Jacobi equation to estimate the basins of attraction of the found eco-evolutionary equilibria and simulate typical eco-evolutionary dynamics of cancer within and outside these estimated basins. While we illustrate our results on the cancer treatment case study, they can be generalized to any situation where a human aims at containing, eradicating, or saving Darwinian systems, such as in managing antimicrobial resistance, fisheries management, and pest management. The obtained results help our understanding of the impact of human interventions and intraspecific competition on the possibility of containing, eradicating, or saving evolving species. This will help us with our ability to control such systems.

## Introduction

Human actions often lead to a rapid evolution in biological entities that humans want to save, contain, or eradicate [1–3]. This rapid evolution may lead these systems to states that are unintended or undesirable. For example, an attempt to eradicate the diamondback moth using different types of insecticides has led to its development of resistance to about 100 active chemicals, putting the diamondback moth first among the 20 most insecticide-resistant insect species [4]. In general, pest resistance as a consequence of the abundant application of pesticides is a well-recognized problem worldwide [5–8]. Similarly, we observe an antibiotic resistance crisis as a consequence of a too extensive antibiotic application [9], decreased fish size followed by a collapse of fisheries due to overfishing [10, 11], and treatment-induced resistance of cancer cells in response to the application of high-dose anticancer treatments [12–17]. In all these examples, human interventions drive the evolution of traits that are undesirable to humans [18–20].

Mathematical models, such as those within evolutionary game theory, can help us understand how different selection pressures imposed by humans affect the evolution of biological systems in terms of their eco-evolutionary dynamics [21–25]. When humans impose their actions on the evolving systems, they become Stackelberg leaders in a game against or for nature. The Stackelberg evolutionary game theory combines Stackelberg and evolutionary game theory and has recently been termed the Stackelberg evolutionary game [19, 20].

While Stackelberg evolutionary game theory focuses on human actions that maximize human own benefit when interacting with evolutionary followers, much less attention has been given to studying how competition between evolutionary followers contributes to human’s ability to bring eco-evolutionary dynamics of these followers to states desired by the human. Here, we will bridge this gap by studying the impact of both the rate of competition between different types of evolutionary followers and the intensity of human action on their ability to stabilize the followers’ eco-evolutionary dynamics.

As a specific case study, we will consider cancer treatment, where the physician applies a constant treatment dose to target a polymorphic cancer cell population consisting of treatment-sensitive and treatment-resistant cancer cells. These cells compete with each other for space and resources, to proliferate and survive [26, 27]. In our modeling, this competition is captured through the carrying capacity and competition matrix, leading to the density- and frequency-dependent selection in cancer cells, respectively.

The resistant cancer cells may evolve more or less resistance in response to both the treatment dose and rate of competition within the cancer cell population. While in early-stage cancer the evolution of resistance may not occur when no resistant cells are present a priori and/or when they are outcompeted by sensitive cells, here we consider a case of advanced cancer where the evolution of resistance is typically inevitable. [28–30]. Our model combines qualitative and quantitative resistance [23]. While we have a distinct resistant population, this population does not have a fixed treatment-induced resistance. Rather, this resistance evolves as a quantitative trait, affected by both the pre-existing and treatment-induced resistant cells.

Our game-theoretic model of cancer treatment is an extension of the evolutionary model introduced by Pressley et al. [31]. Our model incorporates competition between cancer cells to study the impact of competition between cancer cells and the administered treatment dose on the ability to stabilize cancer dynamics.

The rest of this paper is organized as follows: we first introduce our Darwinian dynamics model, which accounts for the effects of both human intervention and intraspecific competition (Sect. 2). Next, we describe the methodology for analyzing equilibria and their stability, estimating basins of attraction, and simulating typical cancer eco-evolutionary trajectories (Sect. 3). We then present the results regarding stability, basins of attraction, and cancer trajectories (Sect. 4). Finally, we discuss the implications of our findings for mathematical oncology and game theory, address limitations, and suggest future research directions (Sect. 5).

## Game-theoretic model

Let us consider a polymorphic eco-evolutionary system modeled through Darwinian dynamics [32–34] where a human imposes selection pressure on this system through a constant action $m\in \mathbb {R}_+^0=\mathbb {R}_{+}\cup \{0\}.$ Here, we are interested in how cancer eco-evolutionary equilibria depend on the treatment dose *m* and competition coefficients $\alpha _{ij}$. For this reason, we do not assume *a priori* any dependence of *m* on state variables in our model. In general, treatment can be time-varying. However, because of our focus on the equilibria, we assume the treatment dose to be constant. The method we use allows for extending this framework to piecewise constant treatment doses.

The population and evolving trait of individuals of type *i* will be denoted by $x_i$ and $u_i,$ respectively, where $\textbf{x}(t)=\left( x_i(t)\right)$ and $\textbf{u}(t)=\left( u_i(t)\right)$ define population and evolving traits of all individuals at time *t*.

Ecological dynamics of individuals of type *i* are defined as

1 $$\begin{aligned} {\dot{x}}_i(t)=x_i(t) G(v(t),\textbf{u}(t),\textbf{x}(t),m)\big |_{v(t)=u_i(t)} \end{aligned}$$

where *G* refers to the fitness-generating function from Darwinian dynamics [32] and *v*(*t*) is the trait of a focal individual of type *i* at time *t*.

Following Fisher’s fundamental theorem of natural selection, the focal evolutionary trait *v*(*t*)  changes in the direction of the fitness gradient $\frac{\partial G}{\partial v}$ [35]. The evolutionary dynamics are defined as follows:

2 $$\begin{aligned} {\dot{u}}_i(t)= \sigma _{i} \frac{\partial G(v(t),\textbf{u}(t),\textbf{x}(t),m)}{\partial v(t)}\bigg |_{v(t)=u_i(t)} \end{aligned}$$

The rate at which the trait $u_i(t)$ changes is given by an evolutionary speed $\sigma _{i}$. A measure of how quickly the trait responds to selection gradients, $\sigma _i$, encapsulates the combined effects of genetic variation, mutation rates, and the overall responsiveness of the trait to evolutionary pressures [32]. While this could reflect the trait’s sensitivity to spontaneous genetic changes, in our formulation, $\sigma _i$ is treated as a constant. It thus serves as an aggregate measure of evolutionary responsiveness, encompassing interactions between the trait and the rest of the system dynamics.

In our model, we assume that all individuals’ strategies have the same evolutionary speed, while in reality $\sigma _{i}$’s may depend on other parameters, such as the population size $\textbf{x}$ and/or may be different for different types of individuals [36–38]. Moreover, as long as $\sigma _{i}$ is constant, it does not affect the eco-evolutionary equilibria of the system.

While the eco-evolutionary dynamics (1–2) can define any polymorphic Darwinian system responding to a constant leader’s action *m*,  here we will analyze an example of cancer treatment game. In this example, $m \ge 0$ corresponds to a constant treatment dose applied by a physician and $\textbf{x}(t)=(x_S(t), x_R(t))^{\top }$ is a vector of populations of cancer cells that are sensitive (*S*) and resistant (*R*) to the treatment, respectively, at time *t*.

We assume that only resistant cells have the capacity to evolve resistance $u_R(t)\in \mathbb {R}_+^0$ and that the G-function of these cells has the same form as introduced in [20, 31], i.e.,

3 $$\begin{aligned} G(v(t),\textbf{u}(t),\textbf{x}(t),m) = r(v(t)) \left( 1- \frac{\sum _{j\in \{R,S\}} a(v(t),u_j(t))x_j(t)}{K}\right) -d - \frac{m}{k+b v(t)}\nonumber \\ \end{aligned}$$

with $a(u_{i},u_{j})=\alpha _{ij}$ defining a competition effect of type *j* on type *i*,  where *i*,  $j \in \{ R,S \}$. In (3), the efficacy of the drug is reduced by a focal cell’s resistance *v*(*t*), innate drug immunity *k*, and the benefit *b* of the resistance *v*(*t*) in reducing therapy efficacy. Various in-vitro and in-vivo studies have demonstrated that resistance may incur a cost [39–41]. Therefore, our model includes this cost of resistance, which affects the proliferation rate of resistant cells. We assume that the resistance cost is expressed through the growth rate *r*(*v*(*t*)) of resistant cells, which decreases with increasing resistance, i.e., $r(v(t))=r_{\max } \, e^{-g\,v(t)}.$

In our model, sensitive cells cannot evolve resistance, i.e., $u_S(t)=0$ for all *t*.

In the remainder of this paper, we drop the time symbol *t* for the sake of simplicity and readability of our notation.

The model of cancer eco-evolutionary dynamics then reads as follows:

4 $$\begin{aligned} {\dot{x}}_S&=x_S \left( r_{\max } \left( 1-\frac{\alpha _{SS}x_S+\alpha _{SR}x_R}{K}\right) -d-\frac{m}{k}\right) \nonumber \\ {\dot{x}}_R&=x_R \left( r_{\max } e^{-g\,u_{R}} \left( 1-\frac{\alpha _{RS}x_S+\alpha _{RR}x_R}{K}\right) -d-\frac{m}{k+b\,u_R}\right) \nonumber \\ {\dot{u}}_R&=\sigma \left( -gr_{\max }e^{-gu_{R}}\left( 1-\frac{\alpha _{RR}x_{R}+\alpha _{RS}x_{S}}{K}\right) + \frac{bm}{(k+bu_{R})^2} \right) \end{aligned}$$

The variables and parameters utilized in (4) and their ranges are summarized in Table 1

**Table 1:** Description of variables and parameters utilized in (4), including their values/ranges

Variable/parameters	Description	Value/range
$$\textbf{x}=\left( \begin{array}{c} x_S\\ x_R \end{array}\right) $$	Population of cancer cells	$[0,K]^2$
$u_{S}$	Resistance strategy of sensitive cells	0
$u_{R}$	Resistance strategy of resistant cells	$\mathbb {R}_0^+$
*v*	Resistance strategy of a focal resistant cell	$\mathbb {R}_0^+$
*g*	Magnitude of cost of resistance	$\mathbb {R}_0^+$
*K*	Carrying capacity	$\mathbb {R}_0^+$
$\alpha _{ij}$	Competition coefficient	$\mathbb {R}_0^+$
$\sigma$	Evolutionary speed	$\mathbb {R}_0^+$
*k*	Innate resistance	$[1, +\infty ]$
*b*	Magnitude of resistance benefit	$\mathbb {R}_0^+$
*m*	Treatment	$\mathbb {R}_0^+$
$r_{\text {max}}$	Maximum growth rate	$\mathbb {R}_0^+$
*d*	Natural death rate	$\mathbb {R}_0^+$

## Methods

### Stability analysis of eco-evolutionary equilibria

We analyze the stability of eco-evolutionary equilibria of (4) using the Lyapunov indirect method [42]. This entails linearizing the eco-evolutionary dynamics (4) around its equilibria and calculating the eigenvalues of the corresponding Jacobian matrix at those equilibria. If real parts of all eigenvalues of the Jacobian expressed in a given equilibrium are negative, this equilibrium is locally stable. If at least one eigenvalue has a positive real part, the corresponding equilibrium is unstable.

### Estimating basins of attraction

In this study, we adopt the parameter values *K*, *k*, $\sigma$, and *b* as suggested by [31]. The remaining parameters are selected to showcase the full spectrum of equilibria and their stability outcomes for the dynamics described in (4). We summarize all parameters in Table 2.

**Table 2:** Parameters utilized in our simulations and approximation of basins of attraction

Parameter	*g*	*K*	$\alpha _{SS}$	$\alpha _{RR}$	*k*	*b*	*m*	$\sigma$	$r_{\max }$	$\alpha _{SR}$	$\alpha _{RS}$	*d*
Value	0.8	$10^4$	1	1	2	10	0.5	1	0.45 (cases 2,3) 0.35 (case 1)	0.15 (case 2) 1.15 (cases 1,3)	0.2 (case 2) 2 (cases 1,3)	0.01 (cases 2,3) 0.2 (case 1)

When there is a single locally stable equilibrium for the dynamics (4), we estimate its basin of attraction using the level set method [43], following the approach described by Yuan and Li [44]. We rescale the initial conditions to $(x_{S}(0)/K, x_{R}(0)/K, u_{R}(0)),$ so that they are within the range $[0,1]\times [0,1]\times [0,8]$, with other parameter values set as listed in Table 2.^1^

We partition the state space $[0,1]\times [0,1]\times [0,8]$ into a computational grid with a step size of 0.05,  to facilitate numerical calculations. Using this grid, we compute viscosity solutions for the corresponding Hamilton-Jacobi equation by solving it backward in time as described in [44]. To achieve this, we employ Ian Mitchell’s toolbox for level set methods [45] since it is capable of solving time-dependent Hamilton-Jacobi equations in any dimension.

When multiple locally stable equilibria are present, we estimate their basins of attraction within the state space $[0,1]\times [0,1]\times [0,8]$. To achieve this, we randomly select 10000 initial conditions $(x_{S}(0)/K, x_{R}(0)/K, u_{R}(0))$ within this state space and simulate their eco-evolutionary trajectories.

If a trajectory converges to a stable equilibrium, the initial condition is considered within the basin of attraction of that equilibrium. Conversely, if during a simulation $u_{R}$ grows unbounded, it indicates that the initial condition is outside the basin of attraction of the considered stable equilibrium, highlighting regions where the system dynamics lead to instability.

In simulations, we consider three different cases. Case 1 with one locally stable trivial equilibrium, case 2 with one locally stable interior equilibrium, and case 3 with two locally stable fully sensitive and fully resistant equilibria. We approximate the basin of attraction for these cases. Moreover, all our calculations are reported with a precision of 3 significant digits.

### Simulations of typical eco-evolutionary trajectories

Here we illustrate the behavior of trajectories inside and outside the basins of attraction of stable equilibria. We demonstrate how stable equilibria attract trajectories within their basins of attraction and how unstable equilibria repel trajectories outside these basins.

We simulate trajectories governed by (4), starting from various random initial conditions $(x_{S}(0), x_{R}(0), u_{R}(0))$. The parameter values used in these simulations are detailed in Table 2. For simulations, we consider three different cases mentioned before. In addition, all our calculations are reported with a precision of 3 significant digits.

## Results

With substitutions

5 $$\begin{aligned} c_{R}(u_R,m)&=r_{\text {max}}e^{-g\,u_{R}} -d - \frac{m}{k+bu_{R}} , \quad c_{RR}(u_R)= \frac{r_{\text {max}}}{K}e^{-g\,u_{R}} , \nonumber \\ c_{S}(m)&=r_{\text {max}} -d - \frac{m}{k}, \quad c_{SS}= \frac{r_{\text {max}}}{K} \end{aligned}$$

Equations (4) can be rewritten as

6 $$\begin{aligned} {\dot{x}}_{R}&=x_{R}\bigg (c_{R}(u_R,m)-\alpha _{RR}c_{RR}(u_{R})x_{R}-\alpha _{RS}c_{RR}(u_R)x_{S}\bigg ) \nonumber \\ {\dot{x}}_{S}&=x_{S}\bigg (c_{S}(m)-\alpha _{SS}c_{SS}x_{S}- \alpha _{SR}c_{SS}x_{R}\bigg ) \nonumber \\ {\dot{u}}_{R}&=\sigma \bigg ( -g\,r_{\max }e^{-gu_{R}}(1-\frac{\alpha _{RR}x_{R}+\alpha _{RS}x_{S}}{K}) + \frac{b\,m}{(k+bu_{R})^2} \bigg ) \end{aligned}$$

The Jacobian matrix $J(x_{S},x_{R},u_{R})$ of (6) is

7 $$\begin{aligned} \begin{pmatrix} c_{S}(m)-c_{SS}(2\alpha _{SS}x_{S}+\alpha _{SR}x_{R}) &  -\alpha _{SR}x_{S} &  0\\ -\alpha _{RS}c_{RR}(u_R)x_{R} &  c_{R}(u_{R},m)-c_{RR}(u_R)(2\alpha _{RR}x_{R}+\alpha _{RS}x_{S}) &  x_{R} \frac{\partial G_{R}}{\partial u_{R}}\\ \sigma g c_{RR}(u_R)\alpha _{RS} &  \sigma g c_{RR}(u_R)\alpha _{RR} &  \sigma \frac{\partial ^{2}G_{R}}{\partial u_{R}^{2}} \end{pmatrix} \end{aligned}$$

where $$G_{R}$$ denotes $$G(v,\textbf{u},\textbf{x},m)\bigg |_{v=u_{R}}$$.

### Eco-evolutionary equilibria and their stability

We have four different potential equilibria of eco-evolutionary dynamics (6): interior, trivial, fully sensitive, and fully resistant equilibria, which will be analyzed in Sects. 4.1.1, 4.1.2, 4.1.3, and 4.1.4, respectively.

#### Interior equilibrium

If an interior equilibrium $\textbf{x}^{*}=(x_{S}^{*}, x_{R}^{*}, u_{R}^{*})^{\top }$ of (6) exists, it satisfies

8 $$\begin{aligned} x_{R}^{*}(m)&=\frac{1}{\text {D}}\bigg ( -\alpha _{RS}\frac{c_S(m)}{c_{SS}} + \alpha _{SS}\frac{c_{R}(u_{R}^{*}(m),m)}{c_{RR}(u_{R}^{*}(m))} \bigg ) \end{aligned}$$

9 $$\begin{aligned} x_{S}^{*}(m)&=\frac{1}{\text {D}}\bigg ( \alpha _{RR}\frac{c_S(m)}{c_{SS}} - \alpha _{SR}\frac{c_{R}(u_{R}^{*}(m),m)}{c_{RR}(u_{R}^{*}(m))} \bigg ) \end{aligned}$$

10 $$\begin{aligned} u_{R}^{*}(m)&=\frac{-k}{b}+ \frac{-mg+\sqrt{m^2g^2+4mgdb}}{2bdg} \end{aligned}$$

where

$$\begin{aligned} \text {D}= \alpha _{SS}\alpha _{RR} - \alpha _{SR}\alpha _{RS} \end{aligned}$$

Existence of the interior equilibrium (8–10) requires the following conditions:

1. Condition $$k^{2}dg+kmg < mb, $$ which ensures that $$u_{R}^{*}(m)$$ is positive.
2. Equations (8) and (9) are positive.

Let us analyze when the second condition is satisfied. We will do so by considering two separate assumptions: $D<0$ and $D>0$, as $D=0$ implies no interior equilibrium:

- Case $D>0$. If $c_{S}(m)<0$, $c_{R}(u_{R}^{*}(m),m)$ must be negative, otherwise $x_{S}^{*}(m)$ becomes negative. For (8) and (9) to become positive, the following has to be held:
  $$\begin{aligned}&\alpha _{SS}\frac{c_{R}(u_{R}^{*}(m),m)}{c_{RR}(u_{R}^{*}(m))}> \alpha _{RS}\frac{c_S(m)}{c_{SS}} \implies \frac{\alpha _{RS}}{\alpha _{SS}}> \frac{c_{R}(u_{R}^{*}(m),m)}{c_{S}(m)}e^{gu_{R}^{*}} \nonumber \\&\alpha _{RR}\frac{c_S(m)}{c_{SS}} > \alpha _{SR}\frac{c_{R}(u_{R}^{*}(m),m)}{c_{RR}(u_{R}^{*}(m))} \implies \frac{\alpha _{RR}}{\alpha _{SR}} < \frac{c_{R}(u_{R}^{*}(m),m)}{c_{S}(m)}e^{gu_{R}^{*}} \end{aligned}$$
  This implies $\frac{\alpha _{RR}}{\alpha _{SR}} < \frac{\alpha _{RS}}{\alpha _{SS}},$ and $D<0$, a contradiction to our assumption that $D>0$. Therefore, if $D>0$, $c_{S}(m)$ must be positive to have an interior eco-evolutionary equilibrium of (4).
- Case $D<0$. If $c_{S}(m)<0$, $c_{R}(u_{R}^{*}(m),m)$ has to be negative, otherwise $x_{R}^{*}(m)$ becomes negative. For (8) and (9) to become positive the following has to be held:
  $$\begin{aligned}&\alpha _{SS}\frac{c_{R}(u_{R}^{*}(m),m)}{c_{RR}(u_{R}^{*}(m))}< \alpha _{RS}\frac{c_S(m)}{c_{SS}} \implies \frac{\alpha _{RS}}{\alpha _{SS}}< \frac{c_{R}(u_{R}^{*}(m),m)}{c_{S}(m)}e^{gu_{R}^{*}} \nonumber \\&\alpha _{RR}\frac{c_S(m)}{c_{SS}} < \alpha _{SR}\frac{c_{R}(u_{R}^{*}(m),m)}{c_{RR}(u_{R}^{*}(m))} \implies \frac{\alpha _{RR}}{\alpha _{SR}} > \frac{c_{R}(u_{R}^{*}(m),m)}{c_{S}(m)}e^{gu_{R}^{*}} \end{aligned}$$
  This implies $\frac{\alpha _{RR}}{\alpha _{SR}} > \frac{\alpha _{RS}}{\alpha _{SS}}$ and $D>0$, a contradiction to our assumption that $D<0$. Therefore, if $D<0$, $c_{S}(m)$ has to be positive to have an interior eco-evolutionary equilibrium of (4).

We can conclude that regardless of the sign of *D*, the condition $c_{S}(m)>0$ has to be satisfied for the existence of an interior equilibrium of (4).

Let us investigate local stability properties of the interior equilibrium (8–10). Eigenvalues of Jacobian (7) evaluated at the interior equilibrium (8–10) are:

11 $$\begin{aligned} \lambda _{1,2}&= \frac{-(c_{RR}(u_{R}^{*}(m))\alpha _{RR}x_{R}^{*}(m)+c_{SS}\alpha _{SS}x_{S}^{*}(m))}{2} \pm \frac{S(m)}{2} \end{aligned}$$

12 $$\begin{aligned} \lambda _{3}&= \sigma \frac{\partial ^{2}G}{\partial u_{R}^{2}}\bigg |_{u_{R}=u_{R}^{*}(m)} \end{aligned}$$

where *S*(*m*) is defined as

13 $$\begin{aligned} {\sqrt{(c_{RR}(u_{R}^{*}(m))\alpha _{RR}x_{R}^{*}(m)+c_{SS}\alpha _{SS}x_{S}^{*}(m))^{2} -4c_{SS}c_{RR}(u_{R}^{*}(m))x_{S}^{*}(m)x_{R}^{*}(m) \cdot \text {D}}} \end{aligned}$$

The eigenvalues (11) and (12) are calculated through expressing $c_{S}(m)$ and $c_{R}(u_{R}^{*}(m),m)$ through equations (8) and (9), as follows:

14 $$\begin{aligned} c_S(m)&= c_{SS}\alpha _{SS}x_{S}^{*}(m) + c_{SS}\alpha _{SR}x_{R}^{*}(m) \end{aligned}$$

15 $$\begin{aligned} c_{R}(u_{R}^{*}(m),m)&= c_{RR}(u_{R}^{*}(m))\alpha _{RS}x_{S}^{*}(m) + c_{RR}(u_{R}^{*}(m))\alpha _{RR}x_{R}^{*}(m) \end{aligned}$$

Through comparing equations ${\dot{u}}_{R}=0$ and ${\dot{x}}_{R}=0$, we can rewrite (12) into

16 $$\begin{aligned} \lambda _{3}= \sigma \left( \frac{gbm}{(k+bu_{R}^{*}(m))^2} - \frac{2b^2 m}{(k+bu_{R}^{*}(m))^3} \right) = \frac{\sigma bm\left( g(k+bu_{R}^{*}(m)) - 2b\right) }{(k+bu_{R}^{*}(m))^3} \end{aligned}$$

##### Theorem 1

If the interior equilibrium $\textbf{x}^{*}$ exists, it is locally stable if $D=\alpha _{SS}\alpha _{RR} - \alpha _{SR}\alpha _{RS}>0.$

##### Proof

According to (11), if $\text {D}<0,$ *S*(*m*) defined by (13) becomes larger than $c_{RR}(u_{R}^{*}(m))\alpha _{RR}x_{R}^{*}(m)+c_{SS}\alpha _{SS}x_{S}^{*}(m)$. As a consequence, one of the eigenvalues (11) becomes positive. But if $\text {D}>0,$ *S*(*m*) from (13) becomes less than the term $c_{RR}(u_{R}^{*}(m))\alpha _{RR}x_{R}^{*}(m)+c_{SS}\alpha _{SS}x_{S}^{*}(m)$ and, subsequently, both eigenvalues (11) become negative.

Moreover, from (16), we can imply that if $k+bu_{R}^{*}(m) < \frac{2b}{g},$ the third eigenvalue $\lambda _{3}$ is also negative.

We can see that this is true from  (10), which implies

17 $$\begin{aligned} -k-bu_{R}^{*}(m)= \frac{gm-\sqrt{g^{2}m^{2}+4gdbm}}{2dg} \end{aligned}$$

Adding $\frac{2b}{g}$ to the both sides of (17) yields

18 $$\begin{aligned} \frac{2b}{g} - (k+bu_{R}^{*}(m)) = \frac{4bd+gm-\sqrt{g^{2}m^{2}+4g b d m}}{2dg} \end{aligned}$$

By comparing $4bd+gm$ and $\sqrt{g^{2}m^{2}+4\,g b dm}$, we can see that (18) is positive and, indeed, $k+bu_{R}^{*}(m) < \frac{2b}{g}$ and $\lambda _{3}$ is always negative. Consequently, if the interior equilibrium (8–10) exists and corresponding $\text {D}$ is positive, this equilibrium is locally stable. $\square$

#### Trivial equilibrium

By a trivial equilibrium we mean an equilibrium with $x_S=x_R=0,$ while ${\dot{u}}_R=0,$ i.e.,

19 $$\begin{aligned} -g r_{\max }e^{-gu_{R}} + \frac{bm}{(k+bu_{R})^2} = 0 \end{aligned}$$

Let us refer to $u_R$ solving (19) as $u_{R}^{\diamond }$ and to the trivial equilibrium as to $\textbf{x}^{\diamond },$ where $\textbf{x}^{\diamond }=(0,0,u_{R}^{\diamond })^{\top }.$

Note that  (19) consists of a sum of an exponential function $g r_{\max }e^{-gu_{R}}$ and a reciprocal squared function $\frac{bm}{(k+bu_{R})^2}$. Therefore, depending on the values of parameters *g*,  $r_{\max },$ *b*,  *m*,  and *k*, this equation can have zero, one, or two solutions: If the value of the exponential function at $u_{R}=0$ is greater than or equal to the value of the reciprocal squared function at $u_{R}=0$, equation (19) will have one solution. Otherwise, equation (19) can have up to two solutions, which means we may have more trivial equilibria.

Eigenvalues of the Jacobian (7) evaluated at $x^{\diamond }$ are:

20 $$\begin{aligned} \lambda _{1}&=c_{S}(m) \end{aligned}$$

21 $$\begin{aligned} \lambda _{2}&=c_{R}(u_{R}^{\diamond },m) \end{aligned}$$

22 $$\begin{aligned} \lambda _{3}&= \sigma \frac{\partial ^{2}G}{\partial u_{R}^{2}}\bigg |_{u_{R}=u_{R}^{\diamond }} \end{aligned}$$

Using (19), we can rewrite (22) as follows:

23 $$\begin{aligned} \lambda _{3}= \sigma \bigg ( \frac{gbm}{(k+bu_{R}^{\diamond })^2} - \frac{2b^2 m}{(k+bu_{R}^{\diamond })^3} \bigg ) = \frac{\sigma bm}{(k+bu_{R}^{\diamond })^2} \bigg ( g - \frac{2b}{k+bu_{R}^{\diamond }}\bigg ) \end{aligned}$$

##### Theorem 2

If a trivial equilibrium $\textbf{x}^{\diamond }$ exists and additionally, $c_{S}(m)<0$, $c_{R}(u_{R}^{\diamond },m)<0,$ and $k+bu_{R}^{\diamond } < \frac{2b}{g}$, then $\textbf{x}^{\diamond }$ is locally stable.

##### Proof

The trivial equilibrium $\textbf{x}^{\diamond }$ is locally stable if the eigenvalues (20–22) have negative real parts, implying also that $c_{S}(m)<0$ and $c_{R}(u_{R}^{\diamond },m)<0.$ Equation (23) implies that $\frac{\sigma bm}{(k+bu_{R}^{\diamond })^2} \bigg ( g - \frac{2b}{k+bu_{R}^{\diamond }}\bigg )<0.$ This is equivalent to $k+bu_{R}^{\diamond } < \frac{2b}{g}$. Thus, if $c_{S}(m)<0$, $c_{R}(u_{R}^{\diamond },m)<0$, and $k+bu_{R}^{\diamond } < \frac{2b}{g},$ while equation (19) has at least one solution, the trivial equilibrium will be locally stable. In this case, if the initial state is within the basin of attraction of $\textbf{x}^{\diamond }$, then the corresponding trajectory will converge to $\textbf{x}^{\diamond }$. $\square$

#### Fully sensitive equilibrium

At this equilibrium, $x_{R}=0.$ Let us refer to this equilibrium as $\textbf{x}^{\dagger }=(0,x_{S}^{\dagger },u_R^{\dagger })^{\top }.$ Substitution of $x_{R}=0$ in $\dot{x}_{S}=0$ and $\dot{u}_{R}=0$ yields the following expressions for $x_{S}^{\dagger }$ and $u_{R}^{\dagger }$:

24 $$\begin{aligned} x_{S}^{\dagger }&= \frac{K}{\alpha _{SS}}\left( 1- \frac{1}{r_{\max }}\left( d+\frac{m}{k}\right) \right) \end{aligned}$$

25 $$\begin{aligned} u_{R}^{\dagger }&\in \left\{ u_{R} \in \mathbb {R}^{+} |-g r_{\max }e^{-gu_{R}}\left( 1 - \frac{\alpha _{RS}x_{S}^{\dagger }}{K}\right) + \frac{bm}{(k+bu_{R})^2} = 0 \right\} \end{aligned}$$

A similar argument as in case of (19) holds for (25), implying that (25) can have up to two solutions.

As $x_S^{\dagger }>0,$ it implies

$$\begin{aligned} d+\frac{m}{k} < r_{\max } \end{aligned}$$

In addition, (25) implies:

$$\begin{aligned} g r_{\max }e^{-gu_{R}^{\dagger }}\bigg ( 1 - \frac{\alpha _{RS}x_{S}^{\dagger }}{K}\bigg ) = \frac{bm}{(k+bu_{R}^{\dagger })^2} \end{aligned}$$

which leads to

26 $$\begin{aligned} 1 - \frac{\alpha _{RS}x_{S}^{\dagger }}{K}>0 \end{aligned}$$

By substituting $x_{S}^{\dagger }$ from (24) to (26), we obtain:

$$\begin{aligned} c_{S}(m) < \frac{\alpha _{SS}}{\alpha _{RS}}r_{\max } \end{aligned}$$

Thus, the existence of the fully sensitive equilibrium requires inequality

27 $$\begin{aligned} 0< c_{S}(m) < \frac{\alpha _{SS}}{\alpha _{RS}}r_{\max } \end{aligned}$$

to be held.

Eigenvalues of the Jacobian (7) evaluated at $\textbf{x}^{\dagger }$ are:

28 $$\begin{aligned} \lambda _{1}&=c_{S}(m)-2c_{SS}\alpha _{SS}x_{S}^{\dagger }=-c_{S}(m) \end{aligned}$$

29 $$\begin{aligned} \lambda _{2}&=c_{R}(u_{R}^{\dagger },m)-c_{RR}(u_{R}^{\dagger })\alpha _{RS}x_{S}^{\dagger } \end{aligned}$$

30 $$\begin{aligned} \lambda _{3}&= \sigma \frac{\partial ^{2}G}{\partial u_{R}^{2}}\bigg |_{u_{R}=u_{R}^{\dagger }} \end{aligned}$$

Using equations $\dot{u}_{R}=0$ and $\dot{x}_{R}=0$, we can rewrite (30) in the following way:

31 $$\begin{aligned} \lambda _{3}= \sigma \bigg ( \frac{gbm}{(k+bu_{R}^{\dagger })^2} - \frac{2b^2 m}{(k+bu_{R}^{\dagger })^3} \bigg ) = \frac{\sigma bm}{(k+bu_{R}^{\dagger })^3} \bigg ( g(k+bu_{R}^{\dagger }) - 2b\bigg ) \end{aligned}$$

Now we introduce $\kappa (u_{R},m) = \alpha _{SS} \frac{c_{R}(u_{R},m)}{c_{RR}(u_{R})} - \alpha _{RS} \frac{c_S(m)}{c_{SS}}$. We can see from (8), that $x_{R}^{*}(m)=\frac{1}{\text {D}} \kappa (u_{R}^{*},m)$.

##### Theorem 3

   If $\textbf{x}^{\dagger }$ exists, and additionally $c_S(m)>0,$ $\kappa (u_{R}^{\dagger },m)<0$, and $k+bu_{R}^{\dagger } < \frac{2b}{g}$, then $\textbf{x}^{\dagger }$ is locally stable.

##### Proof

 According to (28), if $c_{S}(m)>0,$ then $\lambda _{1}<0.$

By substituting (24) in (29), we can rewrite $\lambda _{2}$ in the following way:

32 $$\begin{aligned} \lambda _{2}&= c_{R}(u_{R}^{\dagger },m)-c_{RR}(u_{R}^{\dagger })\alpha _{RS}x_{S}^{\dagger } \nonumber \\&= c_{R}(u_{R}^{\dagger },m) - \frac{\alpha _{RS}}{\alpha _{SS}}c_{RR}(u_{R}^{\dagger }) \frac{c_{S}(m)}{c_{SS}} \nonumber \\&= \frac{c_{RR}(u_{R}^{\dagger })}{\alpha _{SS}} \bigg ( \alpha _{SS} \frac{c_{R}(u_{R}^{\dagger }(m),m)}{c_{RR}(u_{R}^{\dagger }(m))} - \alpha _{RS} \frac{c_S(m)}{c_{SS}} \bigg ) \end{aligned}$$

We can see that $\lambda _{2}=\frac{c_{RR}(u_{R}^{\dagger })}{\alpha _{SS}} \kappa (u_{R}^{\dagger },m)$. Thus, if $\kappa (u_{R}^{\dagger },m) <0$, $\lambda _{2}$ is negative.

Equation (31) implies that if $k+bu_{R}^{\dagger } < \frac{2b}{g}$, $\lambda _{3}$ becomes negative. Thus, if the conditions mentioned in the Theorem are satisfied, the fully sensitive equilibrium will become locally stable.

#### Fully resistant equilibrium

At this equilibrium, which we will denote by $\textbf{x}^{\ddagger }=(x_S^{\ddagger },x_R^{\ddagger },u_R^{\ddagger })^{\top },$ $x_{S}^{\ddagger }=0$, while $x_{R}=x_{R}^{\ddagger }$ and $u_{R}=u_{R}^{\ddagger },$ with

33 $$\begin{aligned} x_{R}^{\ddagger }&= \frac{K}{\alpha _{RR}}\bigg ( 1- \frac{1}{r_{\max }e^{-gu_{R}^{\ddagger }}}(d+\frac{m}{k+bu_{R}^{\ddagger }}) \bigg ) \end{aligned}$$

34 $$\begin{aligned} u_{R}^{\ddagger }&\in \Bigl \{ u_{R} \in \mathbb {R}^{+} |-g r_{\max }e^{-gu_{R}}\bigg ( 1 - \frac{\alpha _{RR}x_{R}^{\ddagger }}{K}\bigg ) + \frac{bm}{(k+bu_{R})^2} = 0 \Bigl \} \end{aligned}$$

Positivity of the right-hand side of (33) implies

35 $$\begin{aligned} r_{\max }e^{-gu_{R}^{\ddagger }}> d+\frac{m}{k+bu_{R}^{\ddagger }} \implies c_{R}(u_{R}^{\ddagger },m) >0 \end{aligned}$$

In addition, $\alpha _{RR}$ does not have any effects on the positivity of $u_{R}^{\ddagger },$ since $\alpha _{RR}$ in (34) will be canceled out by itself in (33).

Substituting (33) to (34) yields:

36 $$\begin{aligned} -gd- \frac{gm}{k+bu_{R}^{\ddagger }} + \frac{bm}{(k+bu_{R}^{\ddagger })^2}=0 \end{aligned}$$

Now we introduce the next lemma, which is used for calculating $u_{R}^{\ddagger }$.

##### Lemma 1

Consider the function

37 $$\begin{aligned} \Xi (u_R,m) = -gd- \frac{gm}{k+bu_{R}} + \frac{bm}{(k+bu_{R})^2} \end{aligned}$$

where $\Xi (u_R,m)=0$ for $u_R = u_{R}^{*}(m)$. For $u_{R} < u_{R}^{*}(m)$, the function $\Xi (u_R,m)>0,$ while for $u_{R} > u_{R}^{*}(m)$, $\Xi (u_{R},m)<0$.

##### Proof

By rewriting dynamics of $u_{R}$ as

38 $$\begin{aligned} \dot{u}_{R}=\sigma \bigg ( -g\frac{\dot{x}_{R}}{x_{R}} \underbrace{-gd- \frac{gm}{k+bu_{R}} + \frac{bm}{(k+bu_{R})^2}}_{\Xi (u_R,m)} \bigg ) \end{aligned}$$

at $u_{R}^{*}(m)$ we have:

39 $$\begin{aligned} -gd- \frac{gm}{k+bu_{R}^{*}(m)} + \frac{bm}{(k+bu_{R}^{*}(m))^2}=0 \end{aligned}$$

Thus, at $$u_{R}^{*}(m)$$,

40 $$\begin{aligned} -gd(k+bu_{R}^{*}(m))^{2}- gm(k+bu_{R}^{*}(m)) + bm =0 \end{aligned}$$

Multiplying $\Xi (u_R,m)$ by $u_{R}$ yields:

41 $$\begin{aligned} \Xi (u_R,m) (k+bu_{R})^{2} = -gd(k+bu_{R})^{2}- gm(k+bu_{R}) + bm \end{aligned}$$

Now we can subtract (40) from (41):

$$\begin{aligned} \Xi (u_R,m) (k+bu_{R})^{2} - 0&= -gd(k+bu_{R})^{2}- gm(k+bu_{R}) + bm \\&\; - (-gd(k+bu_{R}^{*}(m))^{2}- gm(k+bu_{R}^{*}(m)) + bm) \end{aligned}$$

After some algebraic manipulations, we obtain:

42 $$\begin{aligned} \Xi (u_R,m) (k+bu_{R})^{2} = -gb(u_{R}-u_{R}^{*}(m)) \Big ( d(2k+bu_{R} + bu_{R}^{*}) + m \Big ) \end{aligned}$$

By comparing both sides of (42), we can see the sign of $\Xi (u_R,m) (k+bu_{R})^{2}$ is opposite to the sign of $u_{R}-u_{R}^{*}(m)$. Consequently, for $u_{R}>u_{R}^{*}(m)$, we get $\Xi (u_R,m)<0$, and for $u_{R} < u_{R}^{*}(m)$, we get $\Xi (u_R,m)>0$. $\square$

According to Lemma 1, and by comparing (36) with (40), we can see that $u_{R}^{\ddagger }=u_{R}^{*}(m)$.

Eigenvalues of the Jacobian (7) evaluated at $\textbf{x}^{\ddagger }$ are:

43 $$\begin{aligned} \lambda _{1}&=c_{S}(m)-c_{SS}\alpha _{SR}x_{R}^{\ddagger } \end{aligned}$$

44 $$\begin{aligned} \lambda _{2}&=c_{R}(u_{R}^{\ddagger },m)-2c_{RR}(u_{R}^{\ddagger })\alpha _{RR}x_{R}^{\ddagger }= -c_{R}(u_{R}^{\ddagger },m) \end{aligned}$$

45 $$\begin{aligned} \lambda _{3}&= \sigma \frac{\partial ^{2}G}{\partial u_{R}^{2}}\bigg |_{u_{R}=u_{R}^{\ddagger }} \end{aligned}$$

Using (34), we can rewrite (45) as follows:

46 $$\begin{aligned} \lambda _{3}= \sigma \bigg ( \frac{gbm}{(k+bu_{R}^{\ddagger })^2} - \frac{2b^2 m}{(k+bu_{R}^{\ddagger })^3} \bigg ) = \frac{\sigma bm}{(k+bu_{R}^{\ddagger })^3} \bigg ( g(k+bu_{R}^{\ddagger }) - 2b\bigg ) \end{aligned}$$

##### Theorem 4

If $\textbf{x}^{\ddagger }$ exists, and additionally $c_{R}(u_{R}^{\ddagger },m) >0$ and $\text {D} \cdot x_{S}^{*}<0,$ then $\textbf{x}^{\ddagger }$ is locally stable.

##### Proof

Equation (43) can be rewritten as

47 $$\begin{aligned} \lambda _{1}&= c_{S}(m)-c_{SS}\alpha _{SR}x_{R}^{\ddagger } \nonumber \\&= c_{SS}\left( \frac{c_S(m)}{c_{SS}} - \frac{\alpha _{SR}}{\alpha _{RR}}K \left( 1- \frac{1}{r_{\max }e^{-gu_{R}^{*}(m)}}\left( d+\frac{m}{k+bu_{R}^{*}(m)}\right) \right) \right) \nonumber \\&= c_{SS}\bigg ( \frac{c_S(m)}{c_{SS}} - \frac{\alpha _{SR}}{\alpha _{RR}} \frac{K}{r_{\max }e^{-gu_{R}^{*}(m)}} c_{R}(u_{R}^{*}(m),m) \bigg ) \nonumber \\&= c_{SS}\bigg ( \frac{c_S(m)}{c_{SS}} - \frac{\alpha _{SR}}{\alpha _{RR}} \frac{c_{R}(u_{R}^{*}(m),m)}{c_{RR}(u_{R}^{*}(m))} \bigg ) \end{aligned}$$

Comparing (47) with (9), we obtain

48 $$\begin{aligned} \lambda _{1}= c_{SS} \frac{\text {D} \cdot x_{S}^{*}}{\alpha _{RR}} \end{aligned}$$

Thus, if $\text {D} \cdot x_{S}^{*}<0,$ then $\lambda _{1}<0.$ Moreover, since $u_{R}^{\ddagger }=u_{R}^{*}(m)$, similarly to what we showed in subsection 4.1.1, we can conclude that $k+bu_{R}^{\ddagger } < \frac{2b}{g}$, and, as a result, $\lambda _{3}$ is always negative. Consequently, if $c_{R}(u_{R}^{\ddagger },m)$ is positive and $\text {D} \cdot x_{S}^{*}$ is negative, all corresponding eigenvalues are negative, and the fully resistant equilibrium is locally stable.

Note that when $D=0$, (47) implies that instead of the sign of $\text {D} \cdot x_{S}^{*}$, we should check the sign of $\frac{c_S(m)}{c_{SS}} - \frac{\alpha _{SR}}{\alpha _{RR}} \frac{c_{R}(u_{R}^{*}(m),m)}{c_{RR}(u_{R}^{*}(m))}$. And when we substitute $c_{SS}$ and $c_{RR}(u_{R}^{*}(m))$ from into the previous term, that turns into $c_S(m)e^{-gu_{R}^{*}} - \frac{\alpha _{SR}}{\alpha _{RR}} c_{R}(u_{R}^{*}(m),m)$.

#### Effect of competition coefficients on different equilibria

Equation (8) and Theorem 1 imply that for $x_{R}^{*}$ to be positive, it is required that $c_{S}(m) >0$ and

49 $$\begin{aligned} \frac{\alpha _{RS}}{\alpha _{SS}} < \frac{c_{R}(u_{R}^{*}(m),m)}{c_{S}(m)}e^{gu_{R}^{*}} \end{aligned}$$

Moreover, (27) implies:

$$\begin{aligned} \frac{\alpha _{RS}}{\alpha _{SS}} < \frac{r_{\max }}{c_{S}(m)} \end{aligned}$$

Further,  (5) leads to

$$\begin{aligned} \frac{c_{R}(u_{R}^{*}(m),m)}{c_{S}(m)}e^{gu_{R}^{*}} < \frac{r_{\max }}{c_{S}(m)} \end{aligned}$$

Thus, when $c_{S}(m)>0$, $\frac{\alpha _{RS}}{\alpha _{SS}}$ determines whether the cancer dynamics have stable fully sensitive equilibria (FSE) or not. If

50 $$\begin{aligned} \frac{c_{R}(u_{R}^{*}(m),m)}{c_{S}(m)}e^{gu_{R}^{*}}< \frac{\alpha _{RS}}{\alpha _{SS}} < \frac{r_{\max }}{c_{S}(m)}, \end{aligned}$$

the dynamics (4) have at least one stable FSE. But, if

51 $$\begin{aligned} \frac{r_{\max }}{c_{S}(m)}< \frac{\alpha _{RS}}{\alpha _{SS}} \end{aligned}$$

the dynamics (4) have no stable FSE. Moreover, (9), and Theorem 1 imply that for $x_{S}^{*}$ to be positive, inequalities $c_{S}(m) >0$ and

52 $$\begin{aligned} \frac{\alpha _{SR}}{\alpha _{RR}} < \frac{c_{S}(m)}{c_{R}(u_{R}^{*}(m),m)}e^{-gu_{R}^{*}} \end{aligned}$$

have to be held. In addition, (47) implies that for the existence of a stable fully resistant equilibrium (FRE), the following has to be held:

53 $$\begin{aligned} \frac{\alpha _{SR}}{\alpha _{RR}} > \frac{c_{S}(m)}{c_{R}(u_{R}^{*}(m),m)}e^{-gu_{R}^{*}} \end{aligned}$$

**Fig. 1:**
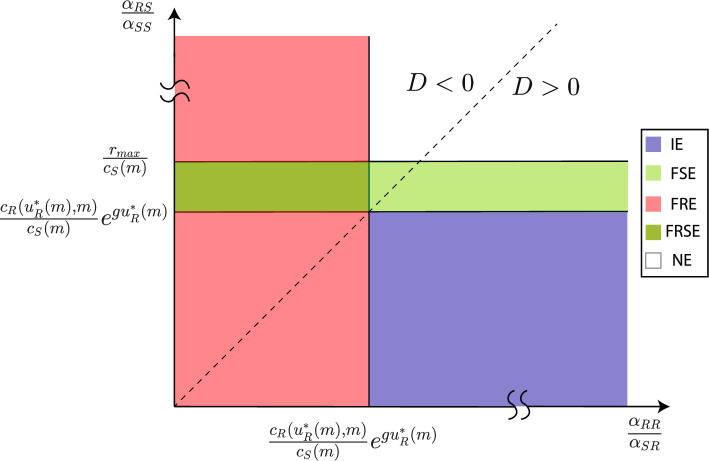
Different types of equilibria in the $(\frac{\alpha _{RS}}{\alpha _{SS}}, \frac{\alpha _{RR}}{\alpha _{SR}})$-plane when $c_{S}(m)$ is positive. Abbreviations: IE - stable interior equilibrium, FSE - stable fully sensitive equilibrium, FRE - stable fully resistant equilibrium, FRSE - stable fully sensitive and fully resistant equilibria, NE - no stable equilibrium

From (49–53), we can determine the type of locally stable equilibria in the $\left( \frac{\alpha _{RS}}{\alpha _{SS}}, \frac{\alpha _{RR}}{\alpha _{SR}}\right)$-plane when $c_{S}(m)>0,$ as demonstrated in Fig. 1.

If $c_{S}(m)<0,$ the signs of $c_{R}(u_{R}^{\diamond },m)$ and $c_{R}(u_{R}^{\ddagger },m)$ determine the existence of the stable trivial and fully resistant equilibria, respectively.

Figure 1 shows that when the net growth rate $c_{S}(m)$ of sensitive cells is positive, the existence of an FRE is dependent on the ratio of competition coefficients $\alpha _{RR}$ and $\alpha _{SR}$. If $\frac{\alpha _{RR}}{\alpha _{SR}}$ is less than $\Gamma = \frac{c_{R}(u_{R}^{*}(m),m)}{c_{S}(m)} e^{g u_{R}^{*}}$, the cancer dynamics will have an FRE. But if $\frac{\alpha _{RR}}{\alpha _{SR}}$ is greater than $\Gamma$, the cancer dynamics can have an IE if $\frac{\alpha _{RS}}{\alpha _{SS}}$ is less than $\Gamma$. The existence of an FSE depends on the ratio of competition coefficients $\alpha _{SS}$ and $\alpha _{RS}$. If $\frac{\alpha _{RS}}{\alpha _{SS}}$ lies between $\frac{r_{\max }}{c_{S}(m)}$ and $\Gamma$, the cancer dynamics will have an FSE. Furthermore, if at the same time $\frac{\alpha _{RR}}{\alpha _{SR}} < \Gamma$, the cancer dynamics will have one FSE and one FRE.

### Estimating basins of attraction

**Case 1**: In this case, we have one locally stable trivial equilibrium. Parameters for this case are mentioned in Table 2. One can obtain:

$$\begin{aligned} x_{S}^{*}&= 1440.000 , \; \; x_{R}^{*}=-2871.000 , \; \; u_{R}^{*}=0.248, \; \; u_{R}^{\diamond } \in \{0.271, 7.135\} \\ \text {D}&= -1.300, \; \; c_{S}(m)=-0.100, \; \; c_{R}(u_{R}^{\diamond },m)\bigg |_{u_{R}^{\diamond }=0.271} = -0.024 \end{aligned}$$

**Fig. 2:**
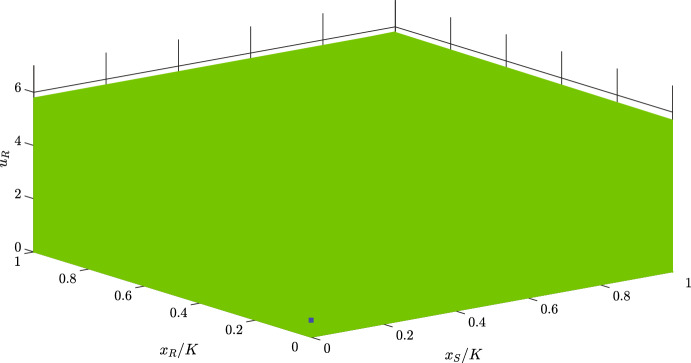
Estimation of the basin of attraction (green) of the locally stable trivial equilibrium (blue). Parameter values: $r_{\max }=0.350$, $g=0.800$, $K=10000$, $\alpha _{SS}=\alpha _{RR}=1$, $\alpha _{SR}=1.150$, $\alpha _{RS}=2$, $d=0.200$, $m=0.500$, $k=2$, $b=10$, and $\sigma =1$

Since $k+bu_{R}^{\diamond }>\frac{2b}{g}$ for $u_{R}^{\diamond }=7.135$, trivial equilibrium $(0,0,7.135)^{\top }$ is unstable. Thus, there exists only one locally stable trivial equilibrium: $(0,0,0.271)^{\top }$ The obtained estimation of the basin of attraction of the locally stable trivial equilibrium is shown in Fig. 2.

**Case 2**: In this case, we approximate the basin of attraction for one locally stable interior equilibrium. Using parameters in Table 2, we obtain

$$\begin{aligned} x_{S}^{*}= 3197.000, \; \; x_{R}^{*}=6833.000, \; \; u_{R}^{*}=0.835 \end{aligned}$$

**Fig. 3:**
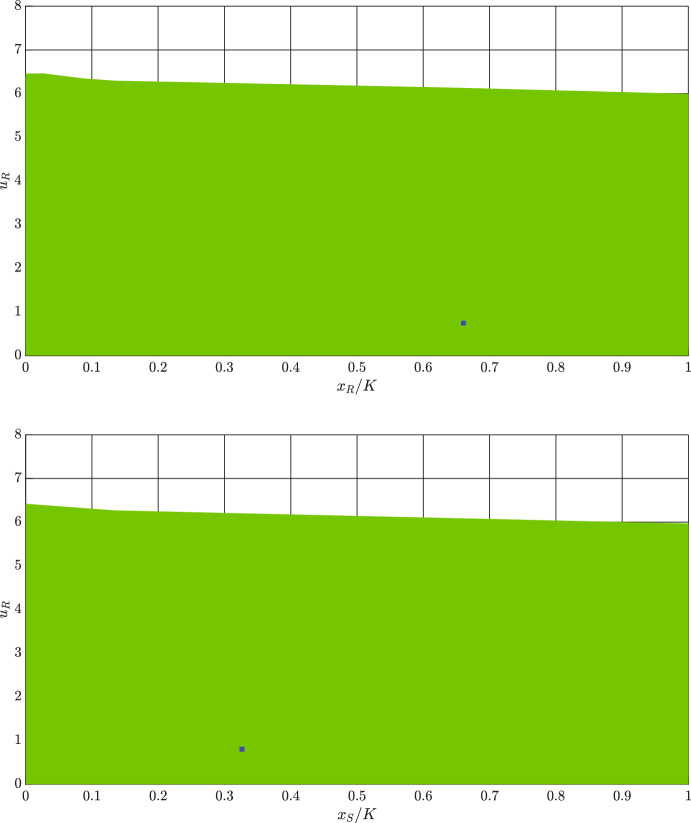
Projections of the basin of attraction (green) of the locally stable interior equilibrium (blue) on the $(x_{S}/K, x_{R}/K)$-and $(x_{R}/K, u_{R})$-planes. Parameter values: $r_{\max }=0.450$, $g=0.800$, $K=10000$, $\alpha _{SS}=\alpha _{RR}=1$, $\alpha _{SR}=0.150$, $\alpha _{RS}=0.200$, $d=0.010$, $m=0.500$, $k=2$, $\sigma =1$, and $b=10$

Since the three-dimensional basin of attraction is easier to see from the two-dimensional projections, we plot such projections on the $\left( x_{S}/K, x_{R}/K\right)$- and $\left( x_{R}/K, u_{R}\right)$-planes in Fig. 3. In these figures, the interior equilibrium is highlighted by a blue square.

From Figs. 2 and 3, we can see that the basins of attraction for both the trivial and interior equilibria are determined by $u_{R}$. This shows that the initial resistance rate is more important than the initial population size of cancer cells for delineating the basin of attraction of a stable equilibrium.

**Case 3**: In this case, dynamics (4) has one fully sensitive and one fully resistant locally stable equilibria. With values from Table 2, we obtain:

$$\begin{aligned} x_{S}^{*}&= 3363.000 , \; \; x_{R}^{*}=747.000 , \; \; u_{R}^{*}=u_{R}^{\ddagger }=0.835 \\ x_{S}^{\dagger }&= 4222.000 , \; \; u_{R}^{\dagger } \in \{1.571, 3.205 \}, \; \; u_{R}^{\diamond } \in \{ 0.204, 7.604 \} \\ x_{R}^{\ddagger }&= 7472.000 , \; \; \text {D}=-1.300, \; \; c_{S}(m)=0.190 \end{aligned}$$

Among the two possible values of $u_{R}^{\dagger }$, only $u_{R}^{\dagger }=1.571$ satisfies $k+bu_{R}^{\dagger }<\frac{2b}{g}$. Thus, $(x_{S}^{\dagger },0, 3.205)^{\top }$ is unstable, while $(x_{S}^{\dagger },0, 1.571)^{\top }$ is locally stable. Moreover, as $c_{R}(u_{R}^{\ddagger },m)$ is positive and $\text {D}.x_{S}^{*}<0$ is negative, $(0,x_{R}^{\ddagger }, u_{R}^{\ddagger })^{\top }$ is locally stable. In the following simulation, we normalize populations $x_i$ of cancer cells $i \in \{R,S\}$ to $x_{i}/K.$

**Fig. 4:**
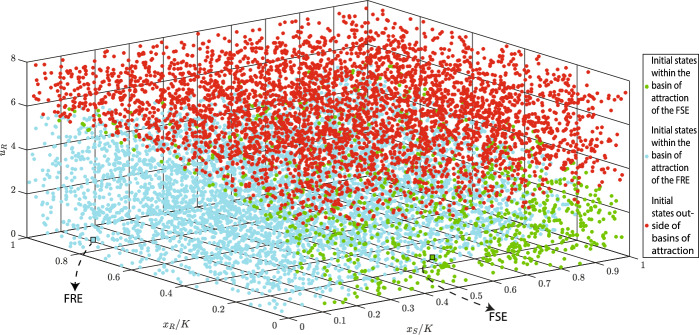
Estimation of basins of attractions of two locally stable equilibria of (1–2). Parameter values: $r_{\max }=0.450$, $g=0.800$, $K=10000$, $\alpha _{SS}=\alpha _{RR}=1$, $\alpha _{SR}=1.150$, $\alpha _{RS}=2$, $d=0.010$, $m=0.500$, $k=2$, $b=10$, and $\sigma =1$. Abbreviations: FRE - fully resistant equilibrium, FSE - fully sensitive equilibrium

**Fig. 5:**
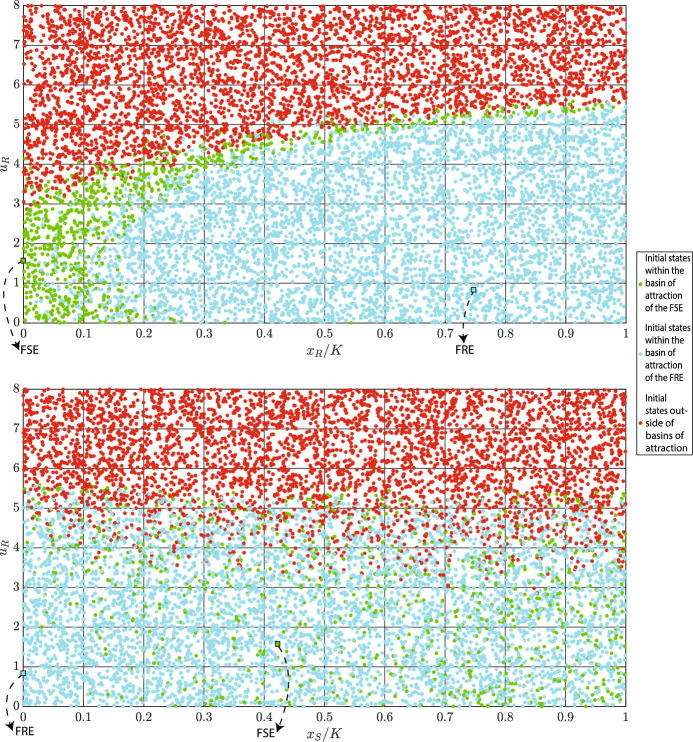
Projections of basins of attraction on the $(x_{S}/K, u_{R})$ and $(x_{R}/K, u_{R})$ planes. Parameter values: $r_{\max }=0.450$, $g=0.800$, $K=10000$, $\alpha _{SS}=\alpha _{RR}=1$, $\alpha _{SR}=1.150$, $\alpha _{RS}=2$, $d=0.010$, $m=0.500$, $k=2$, $b=10$, and $\sigma =1$. Abbreviations: FRE - fully resistant equilibrium, FSE - fully sensitive equilibrium

In Fig. 4, we estimate basins of attractions of the two locally stable equilibria. The fully resistant and fully sensitive equilibria are highlighted by blue and green squares, respectively. Moreover, the basins of attraction of the fully resistant and fully sensitive equilibria are estimated through blue and green dots. Starting from initial conditions highlighted by red dots results in an uncontrollable growth of $u_R$. Projections of the basins of attraction on the two-dimensional planes are shown in Fig. 5.

From Fig. 5, we can see that when cancer dynamics have two stable equilibria, the basin of attraction is determined mainly by $u_{R}$ and $x_{R}$. This shows that the initial resistance rate and the initial population size of resistant cells are more important than the initial population size of sensitive cells for distinguishing the basins of attraction of an FRE and an FSE.

### Simulations of typical cancer trajectories

**Case 1**: In this case, we will see the trajectories that are absorbed by the locally stable trivial equilibrium and those that are repelled from the unstable trivial equilibrium. We saw that we have one locally stable and one locally unstable trivial equilibrium in this case.

**Fig. 6:**
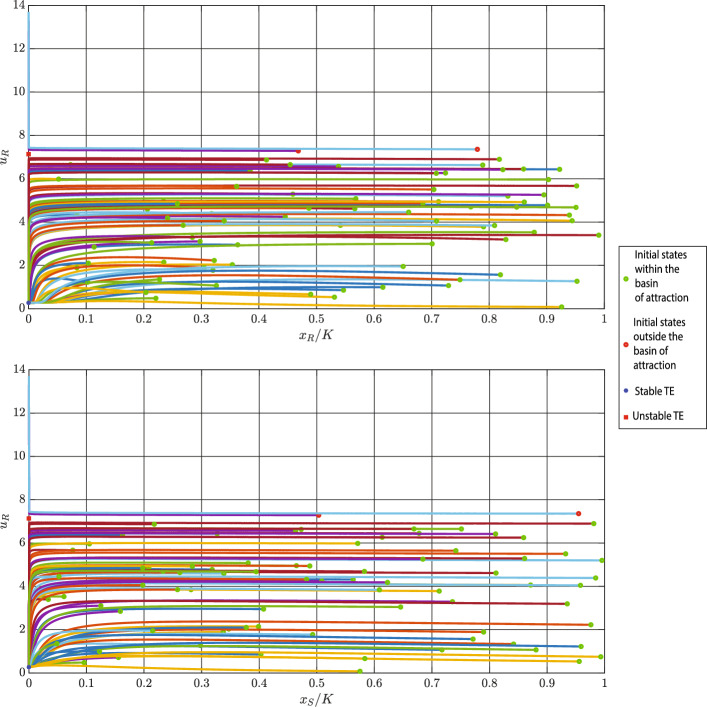
Projections of various eco-evolutionary trajectories starting from different initial conditions on the $(x_{S}/K, u_{R})$-plane and $(x_{R}/K, u_{R})$-plane. In this case, we have two trivial equilibria (TE): The blue star denotes the locally stable equilibrium, while the red square denotes the unstable trivial equilibrium. Parameter values: $r_{\max }=0.35$, $g=0.8$, $K=10000$, $\alpha _{SS}=\alpha _{RR}=1$, $\alpha _{SR}=1.15$, $\alpha _{RS}=2$, $d=0.2$, $m=0.5$, $k=2$, $b=10$, and $\sigma =1$

In Fig. 6, we execute the simulation for 80 different initial conditions. In this figure, the locally stable trivial equilibrium is shown with a blue circle, while the unstable equilibrium is demonstrated with a red square. The initial conditions that end up in the stable equilibrium are illustrated by green circles, while the ones that result in an uncontrollable growth of resistance rate are highlighted by red circles. Moreover, the projections of these trajectories on the $(x_{S}/K, u_{R})$-plane and $(x_{R}/K, u_{R})$-plane are illustrated in Fig. 6.

**Case 2**: As mentioned in Sect. 4.2, in this case, we have one locally stable interior equilibrium. Using parameters in Table 2, we can calculate the following:

$$\begin{aligned}&\frac{x_{S}^{\dagger }}{K}= 0.422 , \; \; \frac{x_{R}^{\ddagger }}{K}=0.747 , \; \; u_{R}^{*}=u_{R}^{\ddagger }=0.835 \\&u_{R}^{\diamond } \in \{0.204, 7.604 \} , \; \; u_{R}^{\dagger } \in \{0.226, 7.441 \}, \;\text {D}=0.970 \end{aligned}$$

**Fig. 7:**
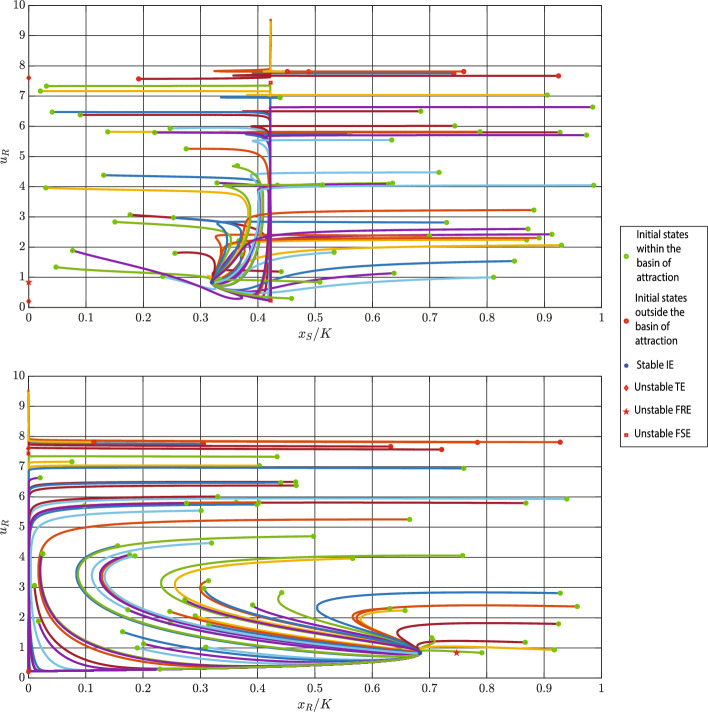
Projection of trajectories starting from different initial conditions on the $(x_{S}/K, u_{R})$- and $(x_{R}/K, u_{R})$-planes. In this case, we have one locally stable interior equilibrium (IE). The blue star denotes the locally stable equilibrium, while the red square, red pentagon, and red diamond denote the unstable fully sensitive (FSE), fully resistant (FRE), and trivial equilibria (TE), respectively. Parameter values: $r_{\max }=0.45$, $g=0.8$, $K=10000$, $\alpha _{SS}=\alpha _{RR}=1$, $\alpha _{SR}=0.15$, $\alpha _{RS}=0.2$, $d=0.01$, $m=0.5$, $k=2$, $b=10$, and $\sigma =1$

In this case, $\text {D}\cdot x_{S}^{*}>0$ and, as a result, the fully resistant equilibrium is unstable. Also, both trivial equilibria are unstable since $c_{S}(m)>0$. Dynamics (4) has two fully sensitive equilibria in this case. Since $\kappa (0.226,m)=0.259 >0$, the equilibrium $(x_{S}^{\dagger },0,0.226)^{\top }$ is unstable. Moreover, for $u_{R}^{\dagger }=7.441$ we have $k+bu_{R}^{\dagger } < \frac{2b}{g}=25$, and, consequently, this trivial equilibrium is unstable, too. Figure 7 demonstrates projections of trajectories starting from 60 different initial conditions on the $(x_{S},K-u_{R})$- and $(x_{R},K-u_{R})$-planes.

From Figs. 6 and 7, we can see that the resistance rate of the unstable trivial equilibrium delineates the border of the basin of attraction of the stable equilibrium.

**Case 3**: In this case, dynamics (4) have one fully sensitive and one fully resistant locally stable equilibria. Using parameter values from Table 2, we obtain:

$$\begin{aligned} x_{S}^{*}&= 3363.000 , \; \; x_{R}^{*}=747.000 , \; \; u_{R}^{*}=u_{R}^{\ddagger }=0.835 \\ x_{S}^{\dagger }&= 4222.000 , \; \; u_{R}^{\dagger } \in \{1.571, 3.205 \}, \; \; u_{R}^{\diamond } \in \{ 0.204, 7.604 \} \\ x_{R}^{\ddagger }&= 7472.000 , \; \; \text {D}=-1.300, \; \; c_{S}(m)=0.190 \end{aligned}$$

In this example, the interior equilibrium exists, but it is unstable, since $\text {D}<0$. Also, $c_{S}(m)>0$, and, as a result, the trivial equilibrium is unstable as well. For $u_{R}^{\dagger }=1.571$, $k+bu_{R}^{\dagger }<\frac{2b}{g}$ and for $u_{R}^{\dagger }=3.205$, $k+bu_{R}^{\dagger }>\frac{2b}{g}$. Thus, $(x_{S}^{\dagger },0, 3.205)^{\top }$ is unstable, while $(x_{S}^{\dagger },0, 1.571)^{\top }$ is locally stable. Moreover, $c_{R}(u_{R}^{\ddagger },m)>0,$ $\text {D}.x_{S}^{*}<0$ and, consequently, $(0,x_{R}^{\ddagger }, u_{R}^{\ddagger })^{\top }$ is locally stable.

**Fig. 8:**
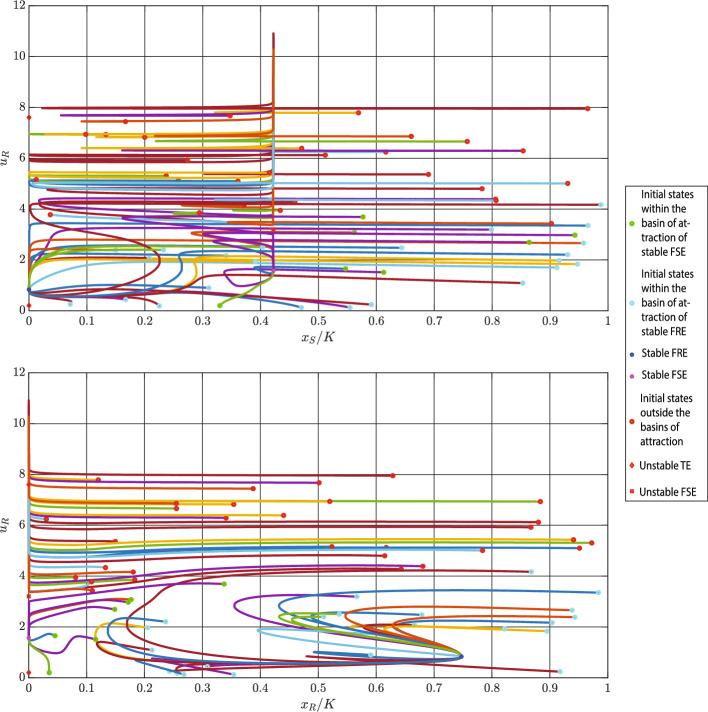
Projection of trajectories starting from different initial conditions on both $(x_{S}/K, u_{R})$- and $(x_{R}/K, u_{R})$-planes. In this case, we have one locally stable fully resistant equilibrium (FRE) and one locally stable fully sensitive equilibrium (FSE). This figure estimates which states are in the basins of attraction of these two locally stable equilibria. Parameter values: $r_{\max }=0.450$, $g=0.800$, $K=10000$, $\alpha _{SS}=\alpha _{RR}=1$, $\alpha _{SR}=1.150$, $\alpha _{RS}=2$, $d=0.010$, $m=0.500$, $k=2$, $b=10$, and $\sigma =1$

In Fig. 8, we demonstrate trajectories started from different initial conditions. One can see the trajectories that are absorbed by both locally stable FSE and locally stable FRE, and those that are repelled by unstable equilibria.

Since we projected three-dimensional trajectories onto two-dimensional planes, some trajectories appear to exhibit sudden changes. However, in the full three-dimensional space, no such sudden changes exist.

## Discussion

In this paper, we analyzed a polymorphic Darwinian dynamics model under human intervention and studied its stability with respect to intraspecific competition and intensity of human intervention. As a specific example of such a Darwinian system, we took cancer under treatment. We analyzed how this system’s eco-evolutionary equilibria depend on competition coefficients and treatment dose. We demonstrated that Darwinian dynamics lead to four different equilibria that can be stable or unstable, depending on the intensity of the human intervention and intraspecific competition. In the cancer treatment case study, we demonstrated that when the treatment dose is sufficiently low to maintain a positive net growth rate of sensitive cells, the system can reach three possible equilibria, depending on the intraspecific competition: (1) interior equilibrium when both effects of sensitive cells on resistant ones and resistant cells on sensitive ones are not too high, (2) fully resistant equilibrium when the effect of resistant cells on sensitive ones is too high, (3) fully sensitive equilibrium when the effect of sensitive cells on resistant ones is neither too high nor too low. Moreover, we showed that when the treatment dose is higher than a threshold, which results in a negative net growth rate of sensitive cells, the system can reach either a fully resistant equilibrium or a trivial equilibrium (extinction of cancer cells) with higher treatment doses. We also approximated basins of attraction of different equilibria using the level-set method. We demonstrated typical eco-evolutionary trajectories of cancer cells within and outside these basins of attraction through simulations.

Our results strengthen our understanding of the stability of evolutionary games. While the stability of different types of dynamic games has been studied extensively [46–51] and also properties of evolutionarily stable equilibria, including their stability, are well defined and understood [34, 52–54], we believe our study is the first one that focuses specifically on the impact of both human intervention and competition coefficients on eco-evolutionary stability. Goh found conditions for globally asymptotically stable equilibria in Lotka-Volterra dynamics [55]. The most closely related work to ours is that of Mougi who added evolutionary dynamics to Lotka-Volterra two-species model with an allelopathic interaction and showed under what conditions these dynamics oscillate or stabilize on an equilibrium by depending on the evolutionary speed  [56]. Here, we added evolutionary dynamics to competitive Lotka-Volterra ecological dynamics under human intervention and demonstrated that there are no globally stable equilibria.

Our results contribute to mathematical oncology as we consider cancer treatment as our main case study. Most existing literature in mathematical oncology uses models with qualitative type of resistance, thus explicitly not considering the evolution of resistance [57–65]. This literature often focuses on treatment protocols for containing tumor burden for as long time as possible. The limited mathematical oncology literature that utilizes models similar to the one introduced in this paper, i.e., models considering resistance as a quantitative evolving trait, mostly assumes either a monomorphic cancer population or a polymorphic population with no competition coefficients, which both lead to no interior eco-evolutionary equilibria [23, 31, 66–68]. Such models are often utilized for treatment optimization with respect to various measurable outcomes, such as time to progression or variance of tumor burden. Adding competition to the polymorphic Darwinian dynamics of cancer, one can focus on therapies stabilizing the eco-evolutionary dynamics of cancer at their interior equilibria, in the spirit of ongoing clinical trial in metastatic ovarian cancer (NCT05080556 [69]). Moreover, our model can be extended to allow for double-bind and extinction therapies, which are novel evolutionary therapies currently tested in initial clinical trials (NCT04343365) [70, 71].

Although we focused on cancer treatment as the main application of our work in this manuscript, the same results can be applied to similar case studies such as antibiotic resistance and pest management. For instance, according to our results in pest management, we should never use pesticides extensively unless there exists a stable trivial equilibrium for the dynamic of evolution of the pest population. If no stable trivial equilibrium exists, one should aim at stabilizing pest population at the interior or fully sensitive or resistant equilibria by using fewer pesticides.

How can physicians use our results? If a stable trivial equilibrium exists, a physician can do so by applying a sufficiently high dose of treatment corresponding to that stable trivial equilibrium and can eradicate all cancer cells. If no stable trivial equilibrium exists and sensitive cells do not evolve, a physician should check the existence of a stable fully sensitive equilibrium. If it exists and tumor volume at this equilibrium is tolerable for the patient, the physician should stabilize the tumor on this fully sensitive equilibrium. If no trivial and fully sensitive equilibrium exists, the physician should aim at stabilizing the tumor on the interior or fully resistant equilibrium. This also depends on the tumor burden at these equilibria. If the corresponding tumor burden is not tolerable for the patient or no stable equilibria exist, the physician should consider maximizing time to progression with the current treatment or using multi-drug therapy [72–74].

To make the results of the case study on cancer treatment presented in this paper applicable in the real world, we need to fit our model to patient time-series data, such as volumetric data (e.g., through serum biomarkers or imaging) [75–77] or information on different cancer cell types (e.g., liquid biopsies [78–80]). While for some cancers, such as prostate cancer, such information is easy to find (prostate-specific antigen; [81]), for other cancers, such as non-small cell lung cancer, we rely on imaging and liquid biopsies. Measuring resistance rate may be (nearly) impossible and must be estimated through time-series data, thus availability of such data is a must.

While this paper focused on equilibria of eco-evolutionary dynamics, future research directions should aim at stabilizing the dynamics at desirable equilibria through optimal control, similarly as it was done in simpler models before in [64, 82–84].

Our model of Darwinian dynamics presented here has some limitations, especially as it approximates evolutionary dynamics less well when the distribution of evolutionary traits is multi-modal or, for example, in the case of disruptive selection [85, 86]. Lion et al. have proposed to apply moment closure approximation to solve this problem [87]. Future research should address how different distributions of evolutionary dynamics impact our ability to capture the eco-evolutionary system in question through Darwinian dynamics and/or how to approximate these dynamics more precisely.

## Data Availability

No datasets were generated or analysed during the current.

## Footnotes

1 If $u_{R}(0)$ exceeds 8 for the parameters in Table 2, $u_{R}(t)$ will grow unbounded.
